# Supplementary material for: Ammonia-oxidizing archaea and bacteria differentially contribute to ammonia oxidation in soil under precipitation gradients and land legacy
Source: bioRxiv. 2023 Nov 12:2023.11.08.566028. Preprint. [Version 1] doi: 10.1101/2023.11.08.566028 (PMC10659370; doi:10.1101/2023.11.08.566028)
Supplement: Supplement 8 — Supplementary Figure S1. Correlation analyses between the detection levels of AOO MAGs and various soil variables (soil pH, iron(III) oxide, nitrate, and ammonia). [file media-8.pdf]

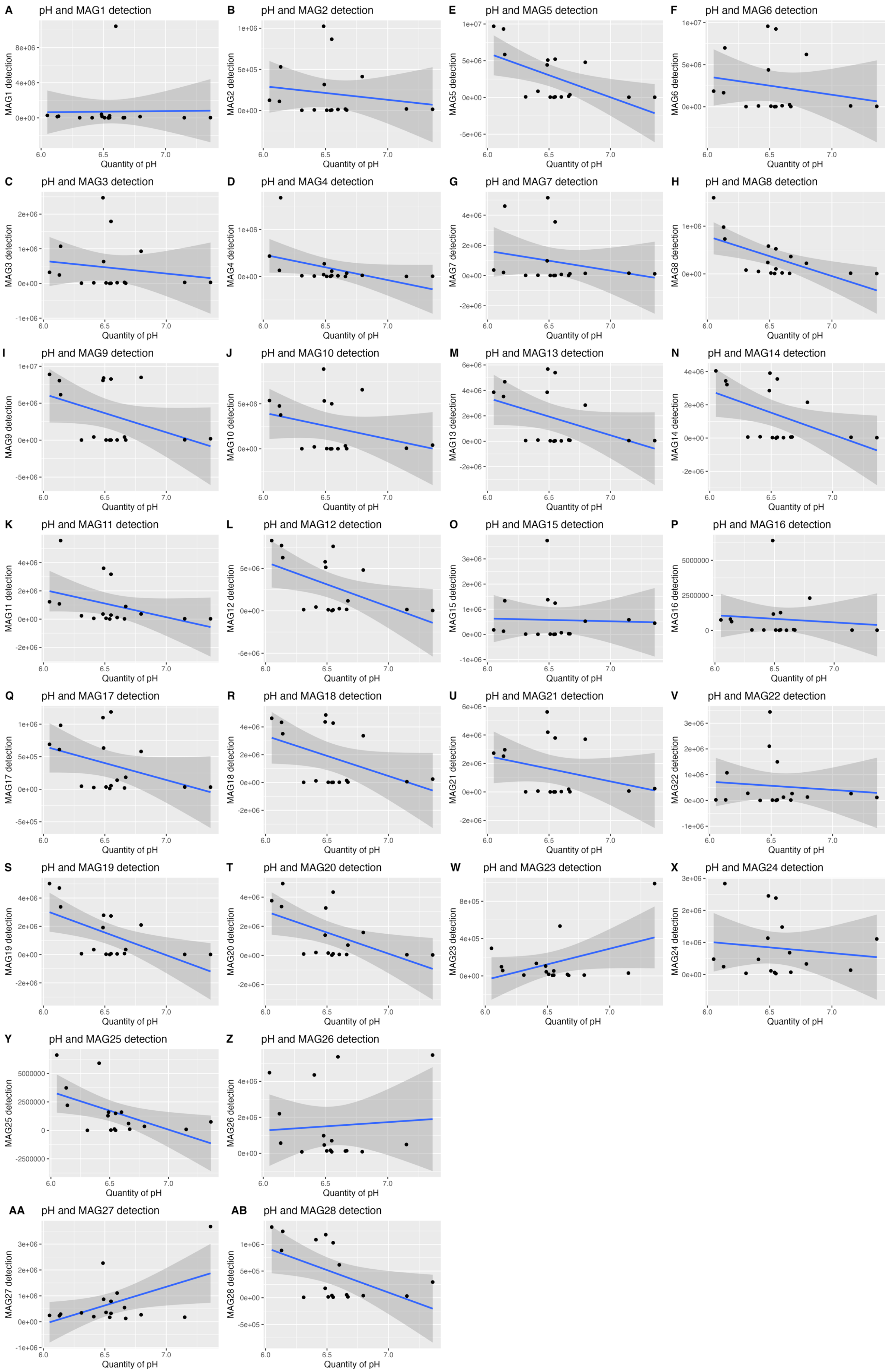
A
pH and MAG1 detection
MAG1 detection
Quantity of pH
B
pH and MAG2 detection
MAG2 detection
Quantity of pH
E
pH and MAG5 detection
MAG5 detection
Quantity of pH
F
pH and MAG6 detection
MAG6 detection
Quantity of pH
C
pH and MAG3 detection
MAG3 detection
Quantity of pH
D
pH and MAG4 detection
MAG4 detection
Quantity of pH
G
pH and MAG7 detection
MAG7 detection
Quantity of pH
H
pH and MAG8 detection
MAG8 detection
Quantity of pH
I
pH and MAG9 detection
MAG9 detection
Quantity of pH
J
pH and MAG10 detection
MAG10 detection
Quantity of pH
M
pH and MAG13 detection
MAG13 detection
Quantity of pH
N
pH and MAG14 detection
MAG14 detection
Quantity of pH
K
pH and MAG11 detection
MAG11 detection
Quantity of pH
L
pH and MAG12 detection
MAG12 detection
Quantity of pH
O
pH and MAG15 detection
MAG15 detection
Quantity of pH
P
pH and MAG16 detection
MAG16 detection
Quantity of pH
Q
pH and MAG17 detection
MAG17 detection
Quantity of pH
R
pH and MAG18 detection
MAG18 detection
Quantity of pH
U
pH and MAG21 detection
MAG21 detection
Quantity of pH
V
pH and MAG22 detection
MAG22 detection
Quantity of pH
S
pH and MAG19 detection
MAG19 detection
Quantity of pH
T
pH and MAG20 detection
MAG20 detection
Quantity of pH
W
pH and MAG23 detection
MAG23 detection
Quantity of pH
X
pH and MAG24 detection
MAG24 detection
Quantity of pH
Y
pH and MAG25 detection
MAG25 detection
Quantity of pH
Z
pH and MAG26 detection
MAG26 detection
Quantity of pH
AA
pH and MAG27 detection
MAG27 detection
Quantity of pH
AB
pH and MAG28 detection
MAG28 detection
Quantity of pH

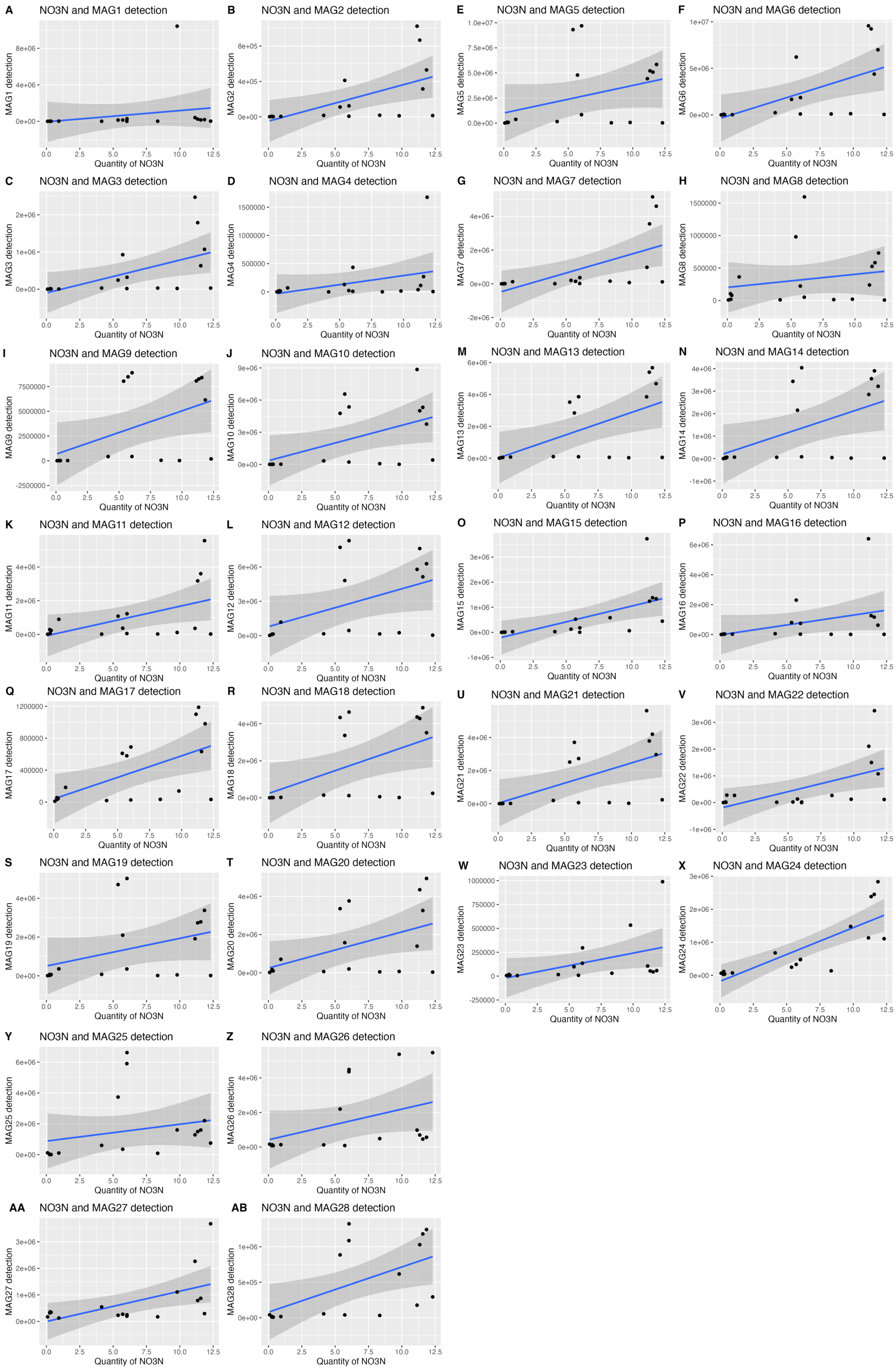
A NO3N and MAG1 detection
B NO3N and MAG2 detection
E NO3N and MAG5 detection
F NO3N and MAG6 detection
C NO3N and MAG3 detection
D NO3N and MAG4 detection
G NO3N and MAG7 detection
H NO3N and MAG8 detection
I NO3N and MAG9 detection
J NO3N and MAG10 detection
M NO3N and MAG13 detection
N NO3N and MAG14 detection
K NO3N and MAG11 detection
L NO3N and MAG12 detection
O NO3N and MAG15 detection
P NO3N and MAG16 detection
Q NO3N and MAG17 detection
R NO3N and MAG18 detection
U NO3N and MAG21 detection
V NO3N and MAG22 detection
S NO3N and MAG19 detection
T NO3N and MAG20 detection
W NO3N and MAG23 detection
X NO3N and MAG24 detection
Y NO3N and MAG25 detection
Z NO3N and MAG26 detection
AA NO3N and MAG27 detection
AB NO3N and MAG28 detection
Quantity of NO3N

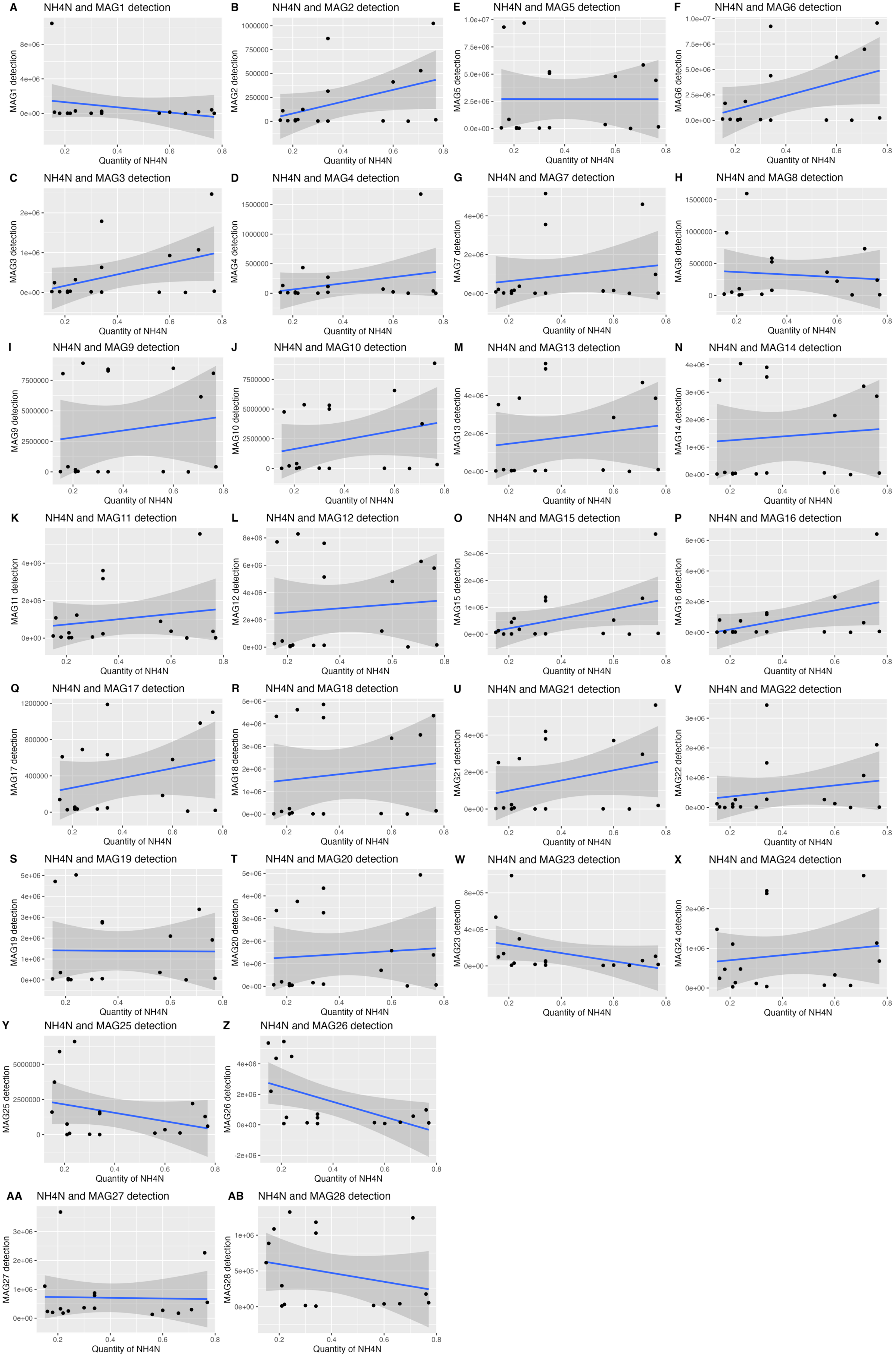
A NH4N and MAG1 detection
B NH4N and MAG2 detection
E NH4N and MAG5 detection
F NH4N and MAG6 detection
C NH4N and MAG3 detection
D NH4N and MAG4 detection
G NH4N and MAG7 detection
H NH4N and MAG8 detection
I NH4N and MAG9 detection
J NH4N and MAG10 detection
M NH4N and MAG13 detection
N NH4N and MAG14 detection
K NH4N and MAG11 detection
L NH4N and MAG12 detection
O NH4N and MAG15 detection
P NH4N and MAG16 detection
Q NH4N and MAG17 detection
R NH4N and MAG18 detection
U NH4N and MAG21 detection
V NH4N and MAG22 detection
S NH4N and MAG19 detection
T NH4N and MAG20 detection
W NH4N and MAG23 detection
X NH4N and MAG24 detection
Y NH4N and MAG25 detection
Z NH4N and MAG26 detection
AA NH4N and MAG27 detection
AB NH4N and MAG28 detection
Quantity of NH4N
MAG1 detection
MAG2 detection
MAG3 detection
MAG4 detection
MAG5 detection
MAG6 detection
MAG7 detection
MAG8 detection
MAG9 detection
MAG10 detection
MAG11 detection
MAG12 detection
MAG13 detection
MAG14 detection
MAG15 detection
MAG16 detection
MAG17 detection
MAG18 detection
MAG19 detection
MAG20 detection
MAG21 detection
MAG22 detection
MAG23 detection
MAG24 detection
MAG25 detection
MAG26 detection
MAG27 detection
MAG28 detection

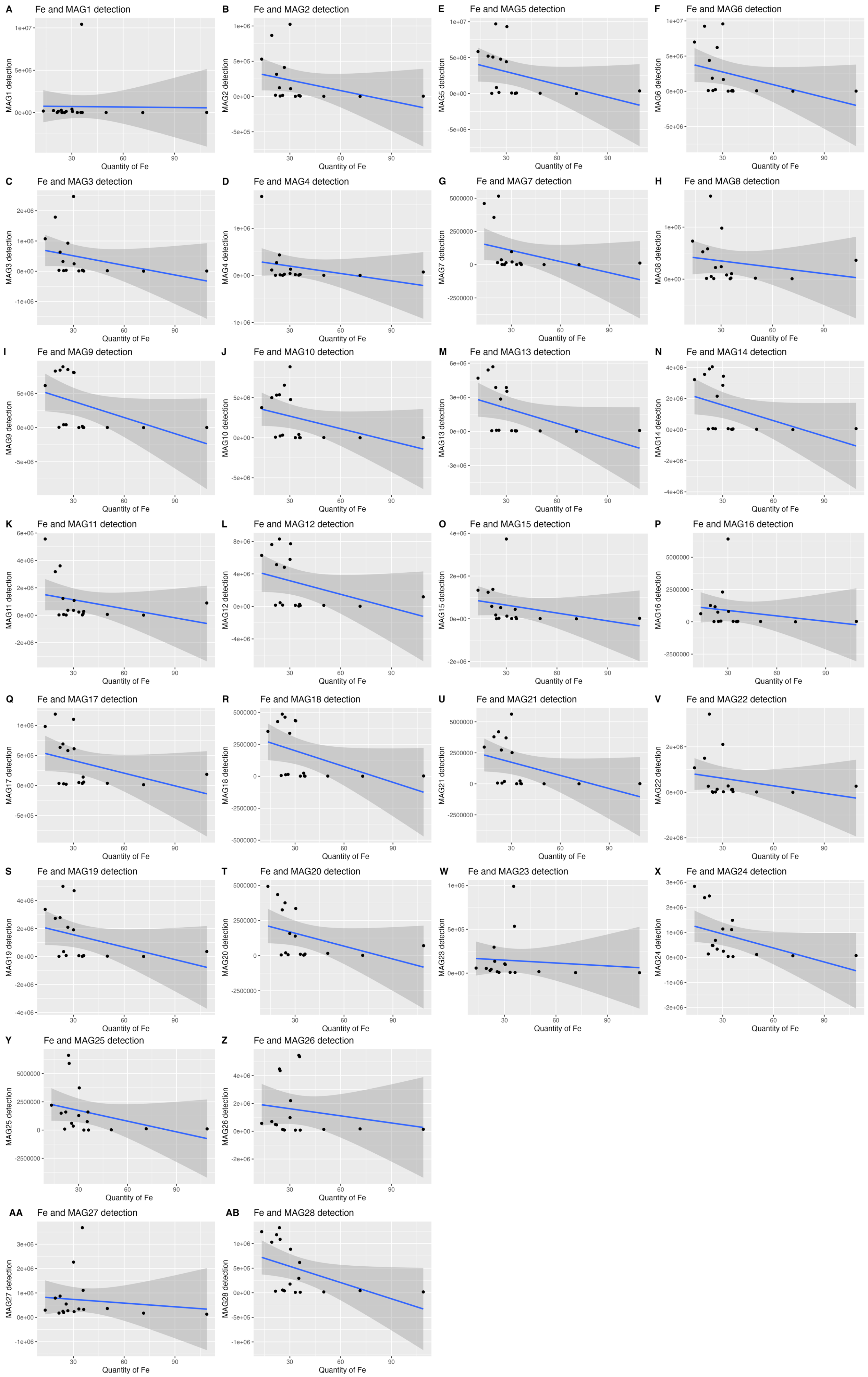

A Fe and MAG1 detection
MAG1 detection
Quantity of Fe
B Fe and MAG2 detection
MAG2 detection
Quantity of Fe
E Fe and MAG5 detection
MAG5 detection
Quantity of Fe
F Fe and MAG6 detection
MAG6 detection
Quantity of Fe
C Fe and MAG3 detection
MAG3 detection
Quantity of Fe
D Fe and MAG4 detection
MAG4 detection
Quantity of Fe
G Fe and MAG7 detection
MAG7 detection
Quantity of Fe
H Fe and MAG8 detection
MAG8 detection
Quantity of Fe
I Fe and MAG9 detection
MAG9 detection
Quantity of Fe
J Fe and MAG10 detection
MAG10 detection
Quantity of Fe
M Fe and MAG13 detection
MAG13 detection
Quantity of Fe
N Fe and MAG14 detection
MAG14 detection
Quantity of Fe
K Fe and MAG11 detection
MAG11 detection
Quantity of Fe
L Fe and MAG12 detection
MAG12 detection
Quantity of Fe
O Fe and MAG15 detection
MAG15 detection
Quantity of Fe
P Fe and MAG16 detection
MAG16 detection
Quantity of Fe
Q Fe and MAG17 detection
MAG17 detection
Quantity of Fe
R Fe and MAG18 detection
MAG18 detection
Quantity of Fe
U Fe and MAG21 detection
MAG21 detection
Quantity of Fe
V Fe and MAG22 detection
MAG22 detection
Quantity of Fe
S Fe and MAG19 detection
MAG19 detection
Quantity of Fe
T Fe and MAG20 detection
MAG20 detection
Quantity of Fe
W Fe and MAG23 detection
MAG23 detection
Quantity of Fe
X Fe and MAG24 detection
MAG24 detection
Quantity of Fe
Y Fe and MAG25 detection
MAG25 detection
Quantity of Fe
Z Fe and MAG26 detection
MAG26 detection
Quantity of Fe
AA Fe and MAG27 detection
MAG27 detection
Quantity of Fe
AB Fe and MAG28 detection
MAG28 detection
Quantity of Fe
